# P47 Condition overlay and diagnostic delay – A challenging case of fibromyalgia and systemic lupus erythematosus

**DOI:** 10.1093/rap/rkae117.078

**Published:** 2024-11-05

**Authors:** Meghna Prabhakar, Marchesa Ataide-Da Costa, Spencer Ellis

**Affiliations:** East and North Hertfordshire NHS Trust, Stevenage, United Kingdom; East and North Hertfordshire NHS Trust, Stevenage, United Kingdom; East and North Hertfordshire NHS Trust, Stevenage, United Kingdom; BSR Education Committee Member, London, United Kingdom

## Abstract

**Introduction:**

Systemic lupus erythematosus (SLE) is an unpredictable disease with significant heterogeneity on presentation. Distinction between disease activity and infection proves challenging. Studies report average delay to diagnosis of 6 years with 47% of patients originally misdiagnosed. Neuropsychiatric SLE (NPSLE) can be a severe manifestation, with up to 40% of neuropsychiatric symptoms developing prior to or at diagnosis. Our case highlights challenges identifying NPSLE, as this patient had a 10-year history of symptoms attributed to fibromyalgia with minimal evidence of active autoimmune disease. This raises the issue of when fibromyalgia may be a misleading label in the context of associated conditions.

**Case description:**

A 43-year-old female collapsed at home, becoming progressively non-verbal with reduced consciousness resulting in intubation and admission to Intensive Care. Collateral history revealed a 6-month duration of lethargy, chest tightness, hair loss, arthralgia, and cough prior to presentation. Her medical history included hypertension, depression and fibromyalgia diagnosed in 2013 with associated Raynaud’s and positive ANA and Anti-Sm autoantibodies. SLE was diagnosed but felt to be clinically inactive whilst fibromyalgia was thought to represent the predominant explanation for her chronic symptoms of headaches and pain exacerbated by psychosocial stressors at the time. The patient was subsequently lost to follow-up post-pandemic. In November 2023, she developed new lymphadenopathy and worsening fatigue leading to re-referral to rheumatology where hydroxychloroquine was started 10-days prior to admission.

Initial hospital management was targeted at infective encephalitis due to high CRP and a low anti-dsDNA level similar to previous clinic values. However, given CSF findings of high protein and lymphocytes with negative septic screens, her neurological decline was subsequently deemed secondary to aseptic meningitis. She developed new renal impairment, thrombocytopenia and anaemia without evidence of haemolysis. These findings, alongside falling complement levels, were ultimately in keeping with NPSLE.

MRI brain showed an acute ischemic stroke in the left frontal lobe and CT thorax and abdomen showed bilateral pneumonia, splenic infarcts and multiple hilar and mediastinal nodes. She had a strongly positive lupus anticoagulant, borderline positive B2 glycoprotein 1 and anticardiolipin antibodies, suggestive of concurrent antiphospholipid syndrome (APS).

Management included ventilatory support, pulsed methylprednisolone, therapeutic anticoagulation, hydroxychloroquine, intravenous antibiotics and antivirals. Following extensive multidisciplinary discussion, intravenous immunoglobulin was administered in preference to cyclophosphamide due to bilateral pneumonia and significant rise in CRP and procalcitonin. Despite these interventions, she remained ventilated via a tracheostomy with poor neurological recovery necessitating therapeutic plasma exchange whilst immunosuppression was considered too high risk.

**Discussion:**

SLE is a multisystem connective tissue disease with broad clinical and laboratory features. The European prevalence is 6.5 to 85 per 100,000. NPSLE can range in presentation from anxiety disorders or psychosis to seizures and cerebral vascular events. The American College of Rheumatology published a standardised nomenclature and case definitions for NPSLE in 1999. This consensus defined 19 neuropsychiatric syndromes of SLE, further classifying them as focal or diffuse. However, as diagnostic criteria are yet to be established, NPSLE remains a diagnosis of exclusion, thereby requiring a high index of suspicion from the treating clinician. This is an important recommendation as her initial diagnosis of fibromyalgia potentially led to misdirection from a diagnosis NPSLE. It is estimated that upwards of 25% of patients presenting with signs of systemic rheumatological conditions such as SLE also fulfil the criteria for fibromyalgia. Given this, there is a possible risk of attributing symptoms of SLE as fibromyalgia if both diagnoses co-exist, as seen in this case.

Given the CSF findings of raised protein and lymphocytes in the absence of microorganisms, a diagnosis of aseptic meningitis was suspected. Aseptic meningitis is an uncommon feature of NPSLE (frequency 0.3-2.7%) whereas cerebral infarcts, as found on this patient’s MRI are more common (frequency 8-15%). It was these findings in the context of rising anti-dsDNA antibodies and falling complement titres that indicated a SLE flare with NPSLE and possible APS. A pregnancy history of preeclampsia was additionally established.

Cyclophosphamide in combination with corticosteroids has shown positive outcomes in SLE. However, it is contraindicated in active infection such as ventilator acquired pneumonia. This case was discussed extensively with the multidisciplinary local and regional teams where intravenous immunoglobulins and plasma exchange was advocated. This has shown benefit in select circumstances although more data exists supporting the use of cyclophosphamide.

**Key learning points:**

• SLE presents with significant heterogeneity and diagnostic delays, especially when coexisting with other conditions, highlighting the importance of a high index of suspicion for timely diagnosis. Given the overlap in symptoms between NPSLE and fibromyalgia, there is risk of attribution if patients have a pre-existing diagnosis of fibromyalgia. In this case, a diagnosis of NPSLE may have been considered at a much earlier stage based on persistent headache and other non-specific symptoms prior to her more serious acute presentation.

• The case also illustrates the therapeutic challenges in managing acute SLE flares and infective complications, including balancing immunosuppressive therapy and infection control particularly in an intensive care setting. Cases complicated by numerous factors underpin the importance of the multidisciplinary team, both locally and regionally where possible. This process may support the consideration of alternative therapeutic regimes when first-line is contraindicated.

• Our case highlights the importance of Anti-Sm antibodies in the diagnostic investigation of SLE. Anti-Sm antibodies hold a low sensitivity but a high specificity for patients with SLE, therefore a positive result makes it very likely that the patient has a diagnosis of SLE. Diagnoses such as fibromyalgia should be made cautiously in cases where Anti-Sm antibody is detected.

